# Supplementary material for: Development and Implementation of a Mobile Application for Choosing Empirical Antimicrobial Therapy for Bacteremia, Pneumonia, Urinary Tract Infection, and Skin and Soft Tissue Infection among Hospitalized Patients
Source: Antibiotics (Basel). 2023 Jan 7;12(1):113. doi: 10.3390/antibiotics12010113 (PMC9855071; doi:10.3390/antibiotics12010113)
Supplement: Supplementary file 1 [file antibiotics-12-00113-s001.zip › antibiotics-2136780-supplementary.pdf]

Supplementary Table S1. Suggested Empiric Antibiotics for Suspected Common Infections in Hospitalized Adult Patients at Siriraj Hospital

**Infection syndrome**	**Risk**	**Suggested Empiric Antibiotic**	**Note**
Sepsis without identified source of infections	CAI, no MDR risk[1]	Ceftriaxone	Add doxycycline or azithromycin if suspected rickettsial infections[2]
	CAI, MDR risk[1]	IV Co-amoxiclav<br>or Piperacillin/tazobactam	Add doxycycline or azithromycin if suspected rickettsial infections[2]
	HAI	Meropenem	Add vancomycin if suspected IV catheter-related infection or evidence of previous MRSA colonization/infection
	HAI, breakthrough carbapenem	Colistin	Add vancomycin if suspected IV catheter-related infection or evidence of previous MRSA colonization/infection
Soft tissue and skin structure infections (SSSIs)	CAI, no MDR risk[1]	IV Cloxacillin or Cefazolin	IV-to-PO switch to dicloxacillin or cephalexin after having clinical improvement and having no contraindication of oral antibiotic
	CAI, MDR risk[1]	Ceftriaxone or<br>IV co-amoxiclav	IV-to-PO switch to co-amoxiclav after having clinical improvement and having no contraindication of oral antibiotic
	CAI, animal bite	IV Co-amoxiclav	IV-to-PO switch to co-amoxiclav after having clinical improvement and having no contraindication of oral antibiotic
	HAI	Piperacillin/tazobactam	Add vancomycin if suspected MRSA infection[3]
		Ceftazidime and clindamycin	Replace clindamycin with vancomycin if suspected MRSA infection[3]
	HAI, breakthrough carbapenem	Colistin and vancomycin	
Pneumonia	CAI, no MDR risk[1]	Ceftriaxone and azithromycin	Replace ceftriaxone with co-amoxiclav if suspected aspiration pneumonia<br>Replace ceftriaxone with ceftazidime if having risk of *Burkholderia pseudomallei* infections<br>Add oseltamivir if having rec recent Flu symptoms or history of close contact to Flu case
	CAI, MDR risk[1]	Ceftriaxone and azithromycin<br>Or levofloxacin monotherapy	Replace ceftriaxone with ceftazidime if having risk of *Burkholderia pseudomallei* infections<br>Add oseltamivir if having recent Flu symptoms or history of close contact to Flu case
	HAI	Piperacillin/tazobactam	Add vancomycin if suspected MRSA infection[3]
		Meropenem	Add vancomycin if suspected MRSA infection[3]
	HAI, breakthrough carbapenem	Colistin	Add vancomycin if suspected MRSA infection[3]<br>Add clindamycin if suspected aspiration pneumonia[4]
Upper UTIs	CAI, no MDR risk[1]	Ceftriaxone	Gentamicin if having normal renal function
	CAI, MDR risk[1]	Amikacin	If having normal renal function
		IV Co-amoxiclav	If having impaired renal function
		Ertapenem	If previous colonization or infection with ESBL-producing GNB in the past 3 months and having impaired renal function
	HAI	Meropenem	
	HAI, breakthrough carbapenem	Colistin	Add vancomycin if suspected MRSA infection[3] or *E. faecium* infection

<u>Abbreviation</u>

CAIs, **community-acquired infections** – the onset of symptoms occurs before hospitalization/within 48 hours after hospitalization and no healthcare associated conditions i.e. chronic hemodialysis, indwelling urinary catheter

HAIs, hospital-acquired infections – the onset of symptoms occurs >48 hours after hospitalization or with healthcare associated conditions i.e. chronic hemodialysis, indwelling urinary catheter

SSSIs, Soft tissue and skin infections – including cellulitis, erysipelas, abscess, surgical site infections, etc.

MDR = Multi-Drug Resistant

MRSA = Methicillin-Resistant *Staphylococcus aureus*

<u>Footnote:</u>

1. **MDR risks** - previous antibiotic therapy or previous MDR bacteria colonization/infection within the past 3 months
2. **Suspected rickettsial infection –** i.e. undifferentiated fever, generalized lymphadenopathy, eschar, history of chigger or tick bite
3. **Suspected MRSA infection** - having evidence of MRSA colonization or gram-staining of clinical specimen shows gram-positive cocci in clusters while receiving beta-lactam therapy
4. **Suspected aspiration pneumonia –** having history of aspiration or having risk of aspiration (i.e. presence of nasogastric tube)
